# A multi-temporal MRI radiomics model for early post-ablation recurrence risk stratification in hepatocellular carcinoma

**DOI:** 10.1016/j.tranon.2026.103014

**Published:** 2026-09-05

**Authors:** Yihang Xu, Liting Peng, Junbin Liao, Zixin Huang, Shiyi Liu, Shuo Wang, Yuxi Huang, Xiaofei Qu, Manxia Lin, Jingxian Shen, Ruiming Liang

**Affiliations:** a Division of Gastrointestinal Surgery, The First Affiliated Hospital, Sun Yat-sen University, Guangzhou, China; b Department of Pathology, The First Affiliated Hospital of Sun Yat-sen University, Guangzhou, China; c Center of Hepato-Pancreato-Biliary Surgery, The First Affiliated Hospital of Sun Yat-sen University, Guangzhou, China; d Division of Interventional Ultrasound, The First Affiliated Hospital of Sun Yat-sen University, Guangzhou, China; e Department of Gastroenterology and Hepatology, The First Affiliated Hospital of Sun Yat-Sen University, Guangzhou, Guangdong, China; f Department of Imaging Diagnosis and Interventional Center, Sun Yat-sen University Cancer Center, Guangzhou, China; g State Key Laboratory of Oncology in South China, Collaborative Innovation Centre for Cancer Medicine, Guangdong Provincial Clinical Research Center for Cancer, Guangdong Esophageal Cancer Institute, Guangzhou, China; h Department of Medical Statistics, Clinical Trials Unit, The First Affiliated Hospital of Sun Yat-Sen University, Guangzhou, China

**Keywords:** Hepatocellular carcinoma, Ablation therapy, Radiomics, Early recurrence, Prognostic model

## Abstract

**Objectives:**

To develop and validate a multi-temporal magnetic resonance imaging (MRI)-based radiomics model for predicting one-year recurrence in hepatocellular carcinoma (HCC) after ablation.

**Methods:**

This retrospective study included 204 patients with HCC who underwent curative ablation between 2016 and 2021. Multi-sequence MRI was performed within one month before and three months after ablation. Radiomics features were extracted from arterial phase (T1A), portal venous phase (T1V), and T2-weighted images using pre-ablation, post-ablation, and delta features. Patients were randomly divided into training (n = 142) and validation (n = 62) cohorts. Models were evaluated using receiver operating characteristic analysis, and differences in AUC between models were assessed.

**Results:**

During a median follow-up of 750 days, 50 patients (24.5%) developed recurrence within one year. The integrated radiomics model (IRM) achieved the best discriminatory performance, with area under the curve values of 0.888 (95% CI: 0.801–0.976) in the training cohort and 0.858 (95% CI: 0.718–0.998) in the validation cohort. Kaplan-Meier analysis showed significant differences in recurrence-free survival between IRM-defined low- and high-risk groups in both cohorts (p < 0.001).

**Conclusion:**

The multi-temporal MRI radiomics model showed promising performance for early recurrence risk stratification after HCC ablation in the internal validation cohort, suggesting potential translational value for non-invasive recurrence-risk assessment. Further prospective external validation is warranted.

## Highlights

• Integrated multi-temporal MRI radiomics model for early HCC recurrence prediction.
• Pre-, post-ablation, and delta MRI features improve risk stratification.
• The model demonstrates strong discrimination performance in validation cohorts.

## Introduction

The global burden of hepatocellular carcinoma (HCC) remains considerable and continues to grow [1]. For patients with early-stage HCC, local ablation is considered a curative treatment option, offering advantages including minimal invasiveness, the possibility of repeated treatment, and preservation of hepatic function [2]. However, early post-ablation tumor recurrence remains the major challenge for HCC management. About 20%−30% of patients recur within one year [3,4]. Early post-ablation recurrence is associated with several clinicopathological and treatment-related factors, including peritumoral microvascular invasion (MVI), satellite nodules, and ablation margin status [[5], [6], [7]]. Therefore, improved assessment of recurrence risk may help optimize surveillance, guide timely intervention, and improve long-term outcomes in patients with HCC.

The superb soft-tissue contrast and multiparametric capabilities of MRI make it a key modality for assessing treatment response in HCC [[8], [9], [10], [11]]. MRI-based radiomics has shown reasonable predictive performance with single-timepoint features [12,13]. For example, Wang et al. constructed a predictive MRI-based model for early postoperative recurrence after curative hepatectomy, achieving an AUC of 0.82 in the validation cohort [14]. In addition, a post-ablation MRI-based radiomics nomogram demonstrated favorable prognostic value for predicting outcomes following locoregional therapy in HCC patients [15,16]. Although previous studies have demonstrated the prognostic value of MRI-based radiomics using pre-treatment or post-treatment imaging alone, the additional value of integrating pre-ablation, post-ablation, and delta radiomics features remains unclear. Because early post-ablation recurrence is influenced by both tumor characteristics and treatment-related factors, longitudinal imaging assessment may provide additional prognostic information beyond single-timepoint analysis [17]. However, the prognostic value of integrating pre-ablation, post-ablation, and delta MRI radiomics features for early recurrence after HCC ablation remains insufficiently validated.

Therefore, this study aimed to develop and evaluate a multi-temporal MRI radiomics model integrating pre-ablation, post-ablation, and delta imaging features for predicting early recurrence after HCC ablation. We further assessed whether the integration of longitudinal imaging information could improve predictive performance compared with single-timepoint radiomics models.

## Methods

### Study population

This retrospective, non-interventional study was approved by the Ethics Committees of The First Affiliated Hospital of Sun Yat-sen University and Sun Yat-sen University Cancer Center, and written informed consent was waived (due to the retrospective nature of the study).

We collected clinical and imaging records from the First Affiliated Hospital of Sun Yat-sen University and Sun Yat-sen University Cancer Center, with all data being anonymized before analysis.

The study retrospectively enrolled HCC patients treated from 2016 to 2021. Inclusion criteria comprised: (1) treatment-naïve patients with pathologically or clinically confirmed HCC; (2) receiving curative local ablation, including radiofrequency ablation (RFA) or microwave ablation (MWA). Exclusion criteria included: (1) prior anti-tumor treatment; (2) absence of standardized contrast-enhanced MRI within one month pre-ablation or three months post-ablation; (3) follow-up duration less than one year; (4) poor MRI image quality inadequate for analysis; and (5) failure to demonstrate complete ablation on the three-month post-ablation MRI, as indicated by radiological evidence of residual viable tumor, recurrence, or imaging findings suspicious for recurrence. Based on these criteria, 204 patients were ultimately enrolled in this study (Fig. 1). The three-month post-ablation MRI served as the landmark assessment, and the primary outcome was recurrence occurring after this landmark and within one year after ablation.

**Fig. 1:**
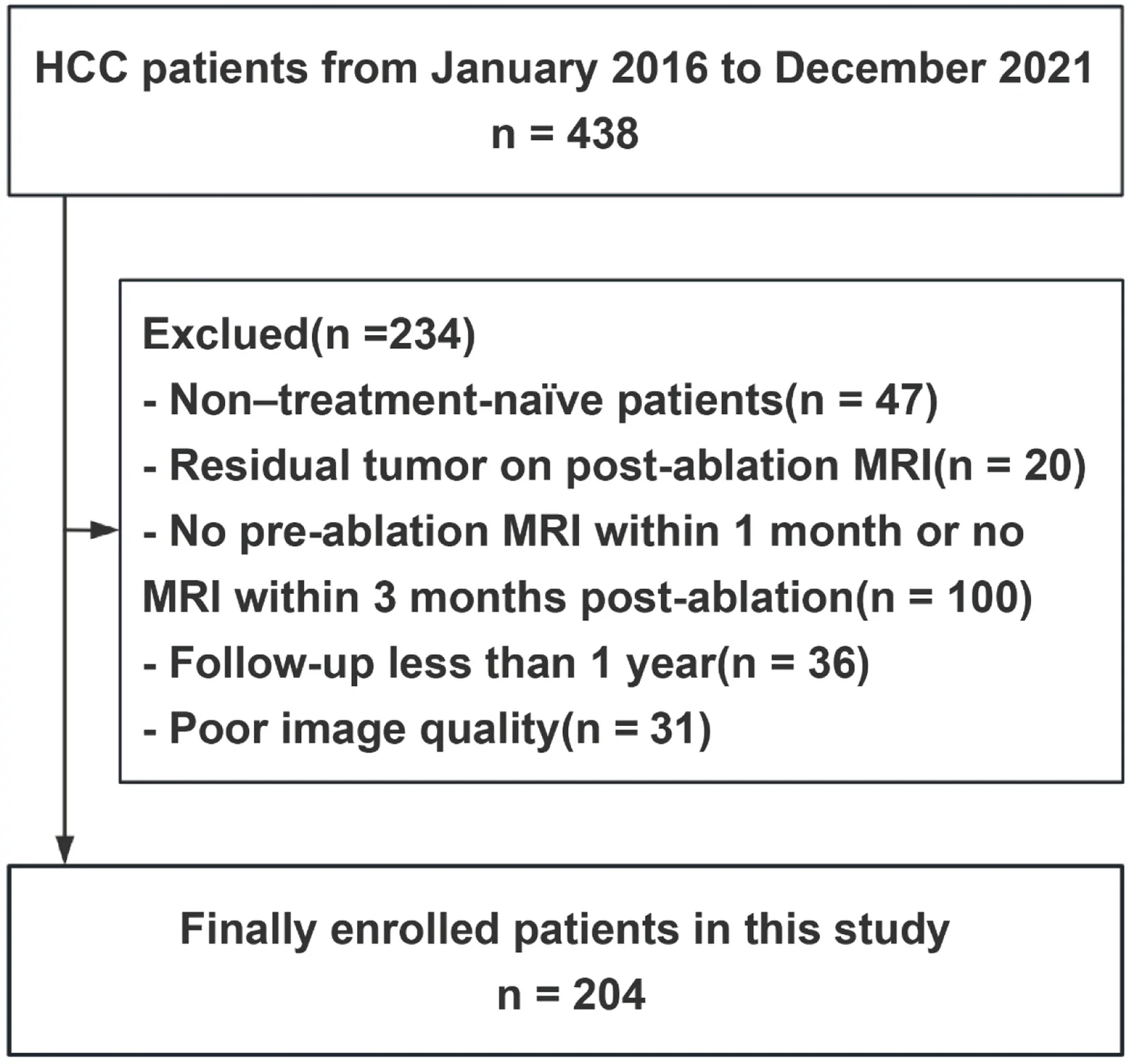
Flowchart of patients included and excluded from this study.

### Ablation procedures and MRI acquisition

All patients underwent real-time ultrasound-guided local ablation therapy performed under the guidance of experienced interventional radiologists. The selection of RFA versus MWA was guided by tumor features and each patient's specific situation. Immediate post-ablation imaging was performed to confirm complete ablation coverage and technical success. Based on routine clinical imaging assessment, all enrolled patients were considered to have an ablation margin greater than 5 mm.

For pre-procedural and follow-up assessment, patients underwent upper abdominal contrast-enhanced MRI within one month before ablation and approximately three months afterward. MRI acquisition included multi-phase sequences comprising T1 arterial phase (T1A), T1 portal venous phase (T1V), and T2-weighted imaging (T2WI) to assess tumor response and surrounding liver parenchyma.

### Delineation of regions of interest (ROIs) and radiomics feature extraction

Regions of interest (ROIs) were delineated on the largest cross-sectional area of T1A, T1V, and T2WI sequences using A.K. software (version 2.0.0; GE Healthcare), with radiomics features extracted from 2D ROIs to ensure reproducibility and clinical feasibility, instead of full 3D segmentation. On pre-ablation MRI, the ROI encompassed the viable tumor, whereas on the three-month post-ablation MRI, the ROI encompassed the corresponding ablation region. Although the paired ROIs represented different biological states, both corresponded to the same treated anatomical site. Two-dimensional ROIs were independently delineated by two experienced radiologists and subsequently reviewed and confirmed by a senior radiologist. From each ROI, 396 radiomics features were automatically extracted using A.K. software. For each MRI sequence, delta features were calculated as the post-ablation feature value minus the corresponding pre-ablation feature value to quantify the net longitudinal imaging change associated with ablation. This approach is consistent with a previously reported longitudinal radiomics strategy that quantified feature differences between corresponding ROIs on serial images [18]. Radiomics feature definitions were consistent with the Image Biomarker Standardisation Initiative (IBSI) recommendations to ensure standardized feature computation.

### Feature selection and model development

Patients were randomly divided into a training cohort (n = 142) and an internal validation cohort (n = 62) at a 7:3 ratio, stratified by outcome to ensure similar recurrence proportions between the two groups. From each MRI sequence, 396 radiomics features were extracted. For the integrated radiomics model (IRM), nine feature sets, comprising pre-ablation (T1A, T1V, and T2WI), post-ablation (T1A, T1V, and T2WI), and delta features (post-ablation minus pre-ablation), were combined, yielding 3564 features per patient. To reduce dimensionality, features with near-zero variance were first removed, followed by correlation-based filtering (Pearson correlation for normally distributed features and Spearman correlation for non-normally distributed features) using an absolute correlation threshold of 0.90. The remaining features were standardized using the mean and standard deviation of the training cohort, and the same parameters were applied to the internal validation cohort. In the training cohort, a random forest algorithm (ntree = 50; mtry = 3) was used to rank feature importance, and the top 10 features were selected for subsequent modeling. A binary logistic regression model was fitted using these 10 features and evaluated in the internal validation cohort (workflow shown in Fig. 2). All preprocessing, feature selection, and model fitting were performed entirely within the training cohort. No cross-validation was performed during model development. To assess the contribution of each temporal component, the same procedure was repeated separately for the pre-ablation, post-ablation, and delta feature sets, yielding the pre-ablation radiomics model (PRM), post-ablation radiomics model (PoRM), and delta radiomics model (DRM), respectively. The IRM was constructed by combining the pre-ablation, post-ablation, and delta feature sets. The performance of all four models was subsequently evaluated in the internal validation cohort.

**Fig. 2:**
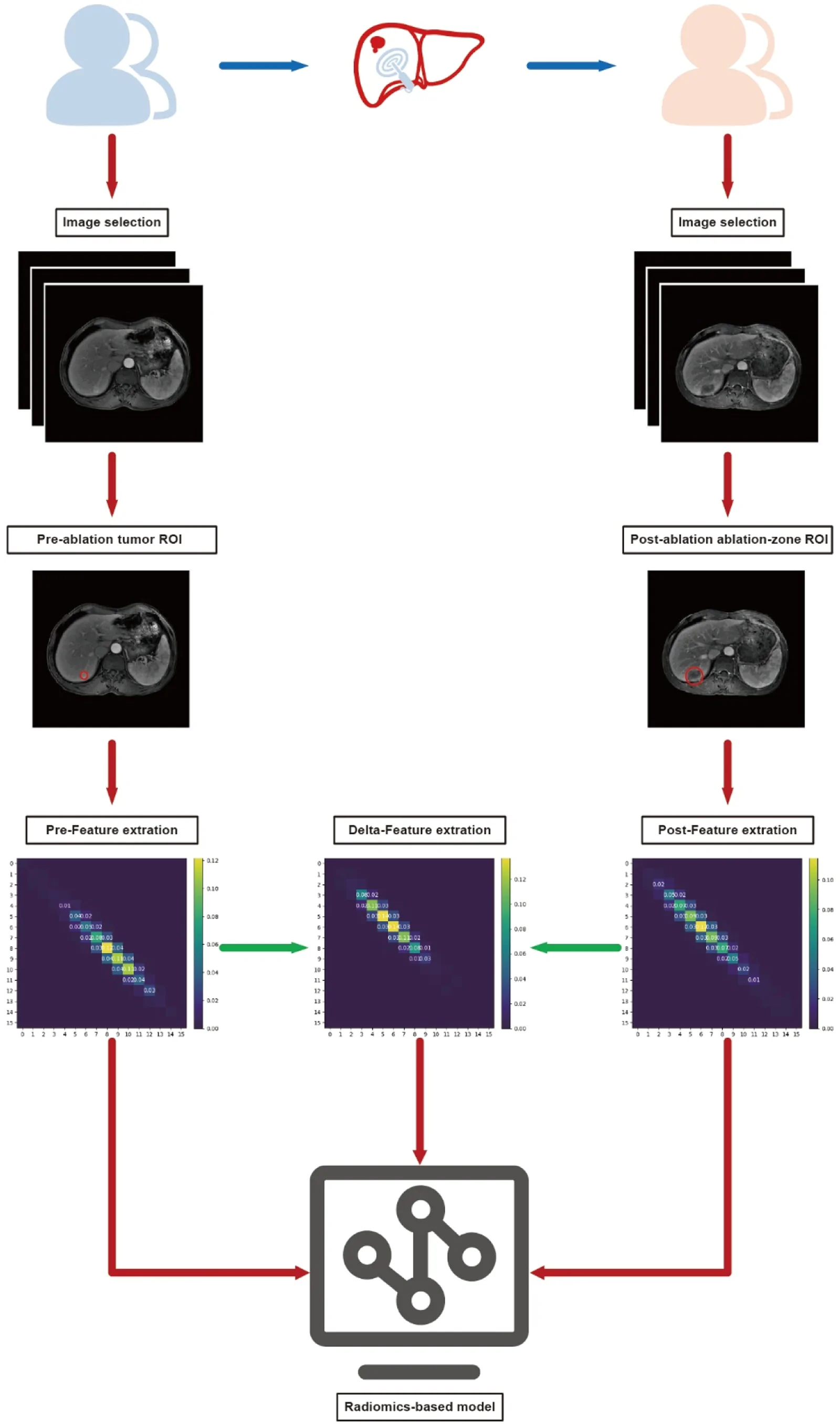
Schematic overview of the radiomics-based workflow for predicting hepatocellular carcinoma recurrence using MRI. The pipeline includes image selection from pre- and post-treatment MRI scans, manual ROI delineation, feature extraction (pre-, delta-, and post-features visualized as heatmaps), and integration into a machine learning model for risk stratification.

### Model comparison

For comparison, a clinical model (CM) was constructed using multivariable logistic regression with 10 prespecified clinical variables: age, sex, tumor size, treatment modality, fatty liver, liver cirrhosis, HBsAg, HBeAg, HBcAb, and AFP. All variables were entered simultaneously into the model without prior univariable screening. A combined model (CRM) was built by integrating the IRM radiomics score with the same 10 clinical variables using logistic regression. For the clinical and combined models, patients with missing clinical variables were excluded, leaving 134 patients in the training cohort and 56 in the validation cohort. To ensure fair comparison across models, the predictive performance of the CM, IRM, and CRM was compared using the same 56-patient internal validation subset. The radiomics models were additionally evaluated in the full 62-patient validation cohort to assess their robustness.

### Risk stratification and survival analysis

The optimal cutoff value of the IRM risk score was determined in the training cohort using the Youden index. The same cutoff was subsequently applied to the internal validation cohort to classify patients into high- and low-risk groups. Recurrence-free survival curves for the IRM-defined risk groups were estimated using the Kaplan-Meier method and compared using the log-rank test. Hazard ratios were estimated using univariable Cox regression.

### Statistical analysis

Continuous variables were compared using Student's *t*-test or the Mann-Whitney U test, and categorical variables using the χ² test or Fisher’s exact test, as appropriate. ROC analysis was used to evaluate model discriminatory performance, with AUCs reported with 95% confidence intervals. Calibration was assessed using calibration curves and Brier scores. Clinical utility was evaluated using decision curve analysis (DCA). For direct comparison, the CM, IRM, and CRM were evaluated in the same validation subset of 56 patients. The IRM was additionally evaluated in the full internal validation cohort of 62 patients. As a separate exploratory analysis, associations between the 10 prespecified clinical variables and recurrence-free survival were examined using a multivariable Cox proportional hazards regression model in the 134 patients with complete clinical data in the training cohort. A two-sided p < 0.05 was considered statistically significant. All analyses were performed using R software (version 4.3.0).

## Results

### Study population characteristics

In total, 204 patients with early-stage HCC who received local ablation therapy were enrolled in this study. Patients’ median age was 54 years (IQR 47–63), and their median follow-up duration was 750 days (IQR 369–1260). During follow-up, 50 patients experienced recurrence within one year, including 35 in the training cohort and 15 in the validation cohort. No significant differences were observed in baseline characteristics between the two cohorts (*p* > 0.05) (Table 1). Recurrence-free survival rates at 1, 2, 3, 4, and 5 years were 75.5%, 60.8%, 54.1%, 44.1%, and 33.3%, respectively. Among the 134 patients with complete clinical data in the training cohort, none of the 10 prespecified clinical variables reached statistical significance in the multivariable Cox regression analysis. The complete results are provided in Supplementary Table S1.

**Table 1:** Baseline characteristics of the study population.

Characteristics	Training cohort	Validation cohort	P Value
	N = 142	N = 62	
Age(years)*	54.08 (11.18)	52.76 (10.38)	0.427
Tumor size(mm)†	19.00 [16.00, 24.00]	20.00 [14.00, 26.75]	0.950
AFP(ng/ml)†	10.00 [4.00, 111.50]	9.00 [4.00, 218.00]	0.592
Gender			
Female	13 (9.2)	6 (9.7)	1.000
Male	129 (90.8)	56 (90.3)	
Ablation			
MWA	56 (39.4)	28 (45.2)	0.677
RFA	85 (59.9)	34 (54.8)	
Missing	1 (0.7)	0 (0.0)	
Adjuvant therapy			
NO	139 (97.9)	58 (93.5)	0.203
YES	3 (2.1)	4 (6.5)	
Fatty liver			
NO	139 (97.9)	60 (96.8)	0.641
YES	3 (2.1)	2 (3.2)	
Liver cirrhosis			
NO	53 (37.3)	20 (32.3)	0.528
YES	89 (62.7)	42 (67.7)	
HBsAg			
Negative	9 (6.3)	8 (12.9)	0.314
Positive	128 (90.1)	52 (83.9)	
Missing	5 (3.5)	2 (3.2)	
HBeAg			
Negative	116 (81.7)	50 (80.6)	0.667
Positive	21 (14.8)	8 (12.9)	
Missing	5 (3.5)	4 (6.5)	
HBcAb			
Negative	3 (2.1)	2 (3.2)	0.570
Positive	134 (94.4)	56 (90.3)	
Missing	5 (3.5)	4 (6.5)	
anti-HCV			
Negative	136 (95.8)	57 (91.9)	0.421
Positive	1 (0.7)	1 (1.6)	
Missing	5 (3.5)	4 (6.5)	
Recurrence within 1 year			
NO	107 (75.4)	47 (75.8)	1.000
YES	35 (24.6)	15 (24.2)	

Other data are presented as n (%).

⁎ Data are presented as mean (SD).

† Data are presented as median [IQR].

### Model performance evaluation

In the training cohort, pre-ablation radiomics model (PRM), post-ablation radiomics model (PoRM), and delta radiomics model (DRM) were constructed based on pre-ablation, post-ablation, and delta features, respectively, to predict one-year recurrence after ablation, achieving an AUC of 0.587, 0.789, and 0.868. Among all models, the integrated radiomics model (IRM) incorporating all three feature types demonstrated favorable discriminatory ability, achieving an AUC of 0.888 (95% CI 0.801–0.976) (Fig. 3A).

**Fig. 3:**
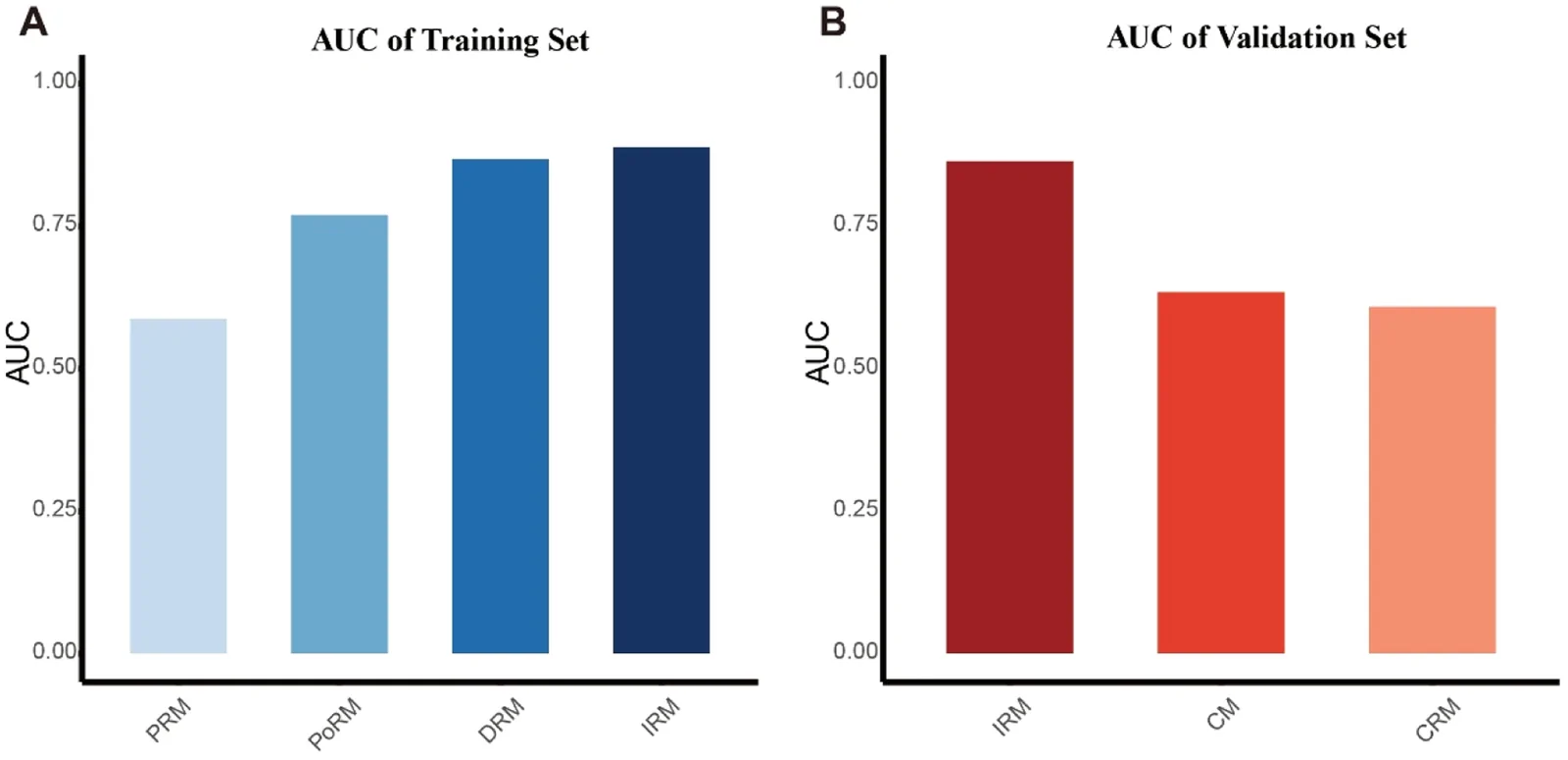
Comparison of model discriminatory performance. (A) AUCs of the pre-ablation radiomics model (PRM), post-ablation radiomics model (PoRM), delta radiomics model (DRM), and integrated radiomics model (IRM) in the training cohort (n = 142). (B) Direct comparison of the clinical model (CM), IRM, and clinical-radiomics combined model (CRM) in the common internal validation subset (n = 56).

In the internal validation cohort, the IRM achieved an AUC of 0.858 (95% CI 0.718–0.998). DeLong test results showed that IRM outperformed PRM (*p* < 0.001) and PoRM (*p* = 0.013) in the validation cohort. At the optimal Youden index threshold, IRM yielded an accuracy of 0.887 (95% CI 0.781–0.953), sensitivity of 0.933 (95% CI 0.681–0.998), and specificity of 0.872 (95% CI 0.743–0.952).

### Model comparison and risk stratification using the IRM

The CM and CRM achieved AUCs of 0.670 (95% CI, 0.570–0.770) and 0.712 (95% CI, 0.611–0.814) in the training cohort, respectively. In the same 56-patient internal validation subset, the AUCs of the CM, IRM, and CRM were 0.621 (95% CI, 0.453–0.789), 0.864 (95% CI, 0.715–1.000), and 0.566 (95% CI, 0.386–0.747), respectively. The IRM had the numerically highest AUC among the three models in this validation subset (Fig. 3B). Calibration curves showed some variability in the internal validation cohort. In the common 56-patient validation subset, the Brier scores were 0.189, 0.197, and 0.201 for the CM, IRM, and CRM, respectively; the IRM yielded a Brier score of 0.192 in the full 62 patient validation cohort. DCA showed overlapping and fluctuating net-benefit curves, with no model demonstrating a consistent advantage across the examined threshold-probability range in the internal validation cohort. Given the limited number of recurrence events, these analyses were considered exploratory. Detailed results are provided in Supplementary Table S2 and Supplementary Figures S1–S2.

To further assess risk-stratification performance, IRM risk scores were used to classify patients into high- and low-risk groups. Patients were stratified into high- and low-risk groups according to the optimal cutoff value derived from the Youden index in the training cohort. Kaplan-Meier survival analysis revealed that the IRM achieved significant stratification of recurrence-free survival (RFS) in both cohorts, with clear separation of survival curves (all log-rank p < 0.001). Median RFS in the training cohort was substantially longer in the low-risk group (52.40 months) than in the high-risk group (4.36 months), corresponding to an HR of 7.26 (95% CI: 4.54–11.60). At 1, 2, and 3 years, recurrence rates were higher in high-risk patients (83.8%, 86.5%, and 93.2%) than in the low-risk group (3.8%, 24.3%, and 35.3%). Consistent results were observed in the validation cohort, with median RFS of 61.43 and 8.47 months in the low- and high-risk groups, respectively (HR = 10.43, 95% CI: 4.66–23.31). In the validation cohort, recurrence rates at 1, 2, and 3 years were 70.0%, 80.0%, and 80.0% in high-risk patients and 2.4%, 14.8%, and 17.5% in low-risk patients (Fig. 4).

**Fig. 4:**
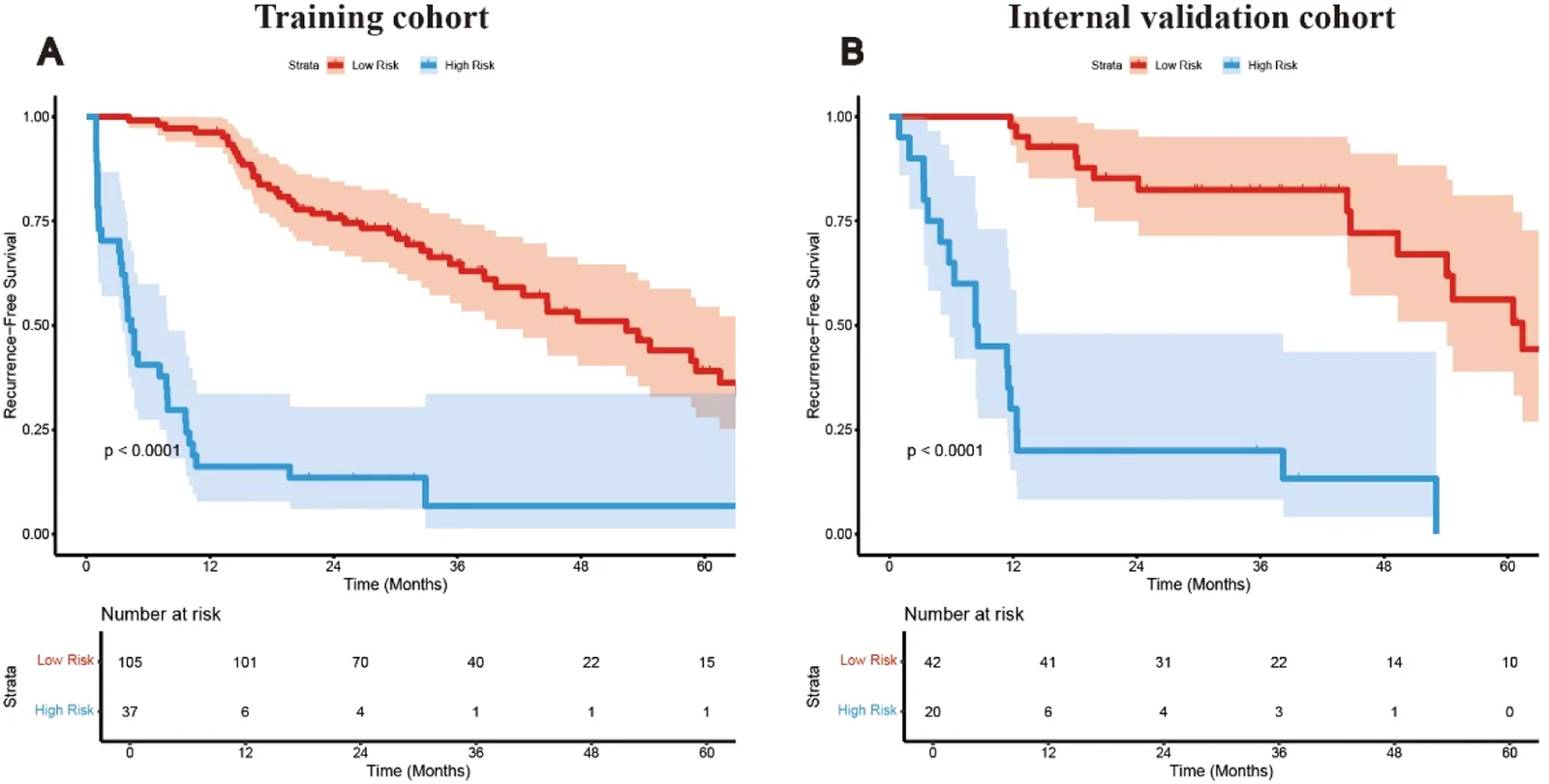
Kaplan-Meier estimates of recurrence-free survival (RFS) in the training and validation cohorts stratified by risk group. Patients were stratified into high-risk (blue) and low-risk (red) groups based on the integrated radiomics model. Log-rank p-values indicate significant differences between risk strata (p < 0.001 for both cohorts). The number at risk is shown below each plot.

### Feature importance and representative cases

Random forest analysis identified the 10 most predictive features primarily derived from the Gray Level Run Length Matrix (GLRLM), including Long Run Low Gray Level Emphasis (LRLGE) and Long Run High Gray Level Emphasis (LRHGE), reflecting tumor internal texture complexity and heterogeneity. Fig. 5 presents two representative cases illustrating differences between conventional clinical assessment and IRM predictions.

**Fig. 5:**
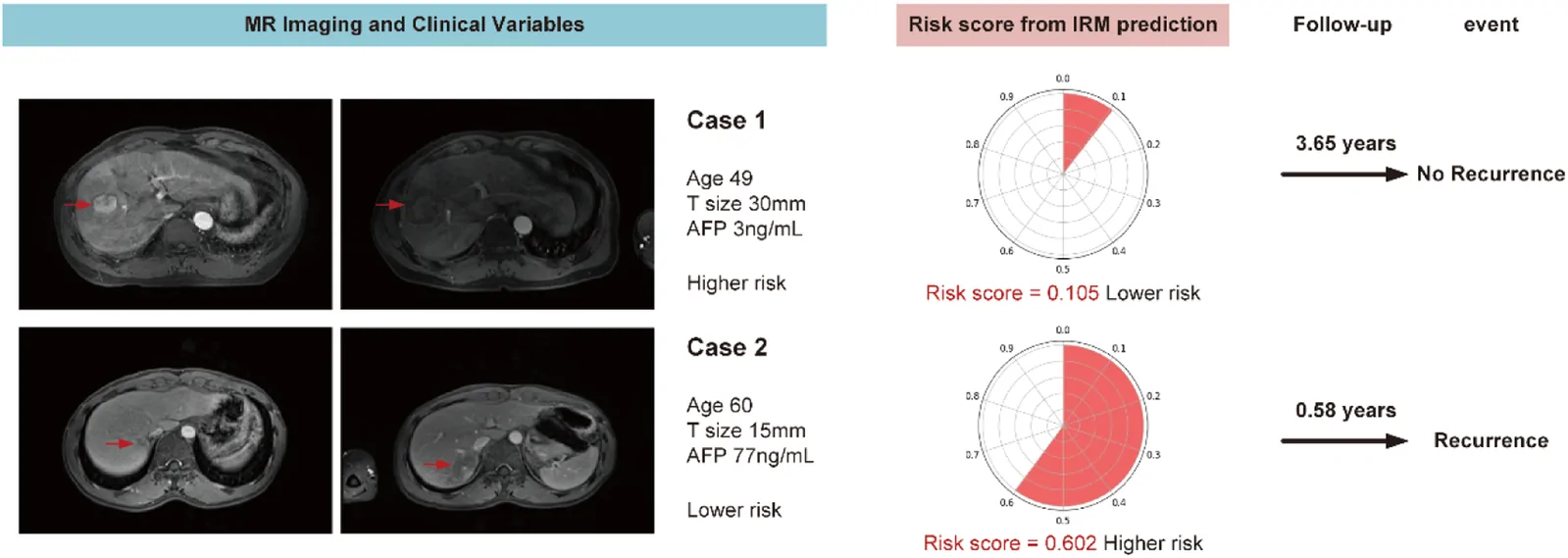
Representative clinical cases illustrating differences between clinical risk assessment and IRM-derived recurrence-risk estimates. Case 1 (age 49 years; tumor size 30 mm; AFP 3 ng/mL; higher clinical risk): Predicted low risk (score = 0.105) with no recurrence at 3.65 years follow-up. Case 2 (age 60 years; tumor size 15 mm; AFP 77 ng/mL; lower clinical risk): Predicted high risk (score = 0.602) with recurrence at 0.58 years follow-up. AFP, alpha-fetoprotein.

## Discussion

This study suggests that a multi-temporal, multi-sequence MRI-based radiomics model may improve early recurrence risk prediction following HCC ablation. By integrating pre-ablation, post-ablation, and delta radiomics features, the model incorporates complementary imaging information that may relate to baseline tumor status, post-ablation imaging changes, and longitudinal variation between imaging time points. The inclusion of delta features provides additional information on imaging changes over time, which may improve recurrence risk stratification. Overall, the integrated radiomics model (IRM) showed favorable discriminatory performance in both the training and internal validation cohorts compared with single-timepoint radiomics and clinical models.

To further investigate the contribution of each temporal component, separate radiomics models were developed based on pre-ablation (PRM), post-ablation (PoRM), and delta (DRM) features, together with an integrated model combining all three feature types. This stepwise framework was designed to assess the incremental value of longitudinal imaging information in recurrence prediction after HCC ablation. PRM may be associated with pre-ablation imaging features, PoRM may be associated with post-ablation imaging changes, and DRM may provide information on imaging variation between pre- and post-ablation states. The IRM integrates these complementary components, offering a more comprehensive imaging-based characterization associated with recurrence risk.

From a feature perspective, the key features identified in this study primarily consist of GLRLM texture features, which high-level radiomics studies have also recognized as tumor recurrence-related characteristics [[19], [20], [21], [22]]. Features such as LRLGE and LRHGE can quantify spatial distribution patterns of tumor internal signal intensity (e.g., necrotic or enhancing regions), reflecting tumor heterogeneity and treatment response. Furthermore, the DRM showed favorable discriminatory performance, suggesting that changes in imaging features between the pre- and post-ablation time points may provide additional prognostic information for recurrence prediction.

Kaplan-Meier curves showed significant differences in RFS between IRM-defined high- and low-risk groups. These findings suggest that IRM-defined risk groups may provide preliminary prognostic information regarding recurrence risk. However, whether the model can inform surveillance or treatment decisions requires confirmation in prospective external cohorts.

This study has several limitations despite its positive findings. First, the retrospective study design may have introduced selection bias. The relatively small sample size (n = 204) and limited number of recurrence events, particularly the 15 events in the internal validation cohort, may have reduced the precision and stability of the performance estimates, as reflected by the wide 95% confidence interval of the validation AUC, and increased the risk of overfitting. Second, the validation was limited to an internal cohort. Future studies with larger external cohorts will be needed to further assess the stability and generalizability of the model. Third, although patients with residual viable tumor, recurrence, or imaging findings suspicious for recurrence on the three-month post-ablation MRI were excluded, subtle subclinical changes not identifiable by routine imaging assessment could not be completely ruled out. Finally, the models incorporated radiomics and clinical variables but did not include pathological or genomic information. Future studies integrating multi-omics data and advanced machine-learning approaches may further improve model performance and interpretability.

In conclusion, this study developed a multi-temporal MRI radiomics model for predicting early recurrence following HCC ablation. The integration of pre-ablation, post-ablation, and delta imaging features may provide additional prognostic information beyond single-timepoint imaging analysis. These findings suggest that longitudinal MRI-based radiomics could serve as a potential non-invasive tool to support risk stratification in patients with HCC after ablation. Further external and prospective studies are required to validate its robustness and clinical applicability.

## Conclusion

This study developed and internally evaluated a multi-temporal, multi-sequence MRI radiomics model for early recurrence risk stratification after HCC ablation. The IRM combined pre-ablation, post-ablation, and delta radiomics features and showed promising discriminatory and risk-stratification performance. These findings suggest the potential translational value of longitudinal MRI radiomics for non-invasive recurrence-risk assessment. Further external prospective studies are warranted to confirm the robustness and generalizability of the model.

## Funding

This study was supported by the Natural Science Foundation of Guangdong Province (Grant No 2023A1515012464) and the National Natural Science Foundation of China (Grant No 82-573-455).

## Institutional review board statement

The study was conducted in accordance with the Declaration of Helsinki. Both institutional ethics committees approved the image data collection and retrospective study protocol (Approval Nos. [2023] 782 and  SZR2019-051). The requirement for written informed consent was waived by the institutional review board owing to the retrospective nature of the study.

## Data availability

The data used in this study are not publicly available due to patient privacy concerns and institutional regulations.

## CRediT authorship contribution statement

**Yihang Xu:** Writing – original draft, Supervision, Project administration, Methodology, Conceptualization. **Liting Peng:** Validation, Investigation. **Junbin Liao:** Validation, Investigation. **Zixin Huang:** Validation. **Shiyi Liu:** Data curation. **Shuo Wang:** Visualization. **Yuxi Huang:** Data curation. **Xiaofei Qu:** Data curation. **Manxia Lin:** Writing – review & editing, Supervision. **Jingxian Shen:** Project administration, Funding acquisition, Conceptualization. **Ruiming Liang:** Project administration, Methodology, Formal analysis.

## Declaration of competing interest

The authors declare that they have no known competing financial interests or personal relationships that could have appeared to influence the work reported in this paper.

## Appendix Supplementary materials

Image, application 1
