# Supplementary material for: A multi-temporal MRI radiomics model for early post-ablation recurrence risk stratification in hepatocellular carcinoma
Source: Transl Oncol. 2026 Sep 5;73:103014. doi: 10.1016/j.tranon.2026.103014 (PMC13679617; doi:10.1016/j.tranon.2026.103014)
Supplement: Supplementary file 1 [file mmc1.docx]

**Supplementary Materials**

**Supplementary Tables**

**Supplementary Table S1.** Multivariable Cox proportional hazards regression analysis of clinical factors associated with recurrence-free survival in the training cohort

| **Variable** | **Adjusted HR (95% CI)** | **P value** |
| --- | --- | --- |
| Age | 1.014 (0.979-1.051) | 0.431 |
| Tumor size | 1.024 (0.983-1.068) | 0.253 |
| AFP | 1.024 (0.843-1.244) | 0.813 |
| **Sex** |  |  |
| Female | Reference | - |
| Male | 1.940 (0.503-7.478) | 0.336 |
| **Treatment modality** |  |  |
| MWA | Reference | - |
| RFA | 0.888 (0.420-1.878) | 0.756 |
| **Fatty liver** |  |  |
| No | Reference | - |
| Yes | 2.647 (0.297-23.582) | 0.383 |
| **Liver cirrhosis** |  |  |
| No | Reference | - |
| Yes | 1.951 (0.818-4.655) | 0.132 |
| **HBsAg** |  |  |
| Negative | Reference | - |
| Positive | 0.972 (0.118-8.030) | 0.979 |
| **HBeAg** |  |  |
| Negative | Reference | - |
| Positive | 1.956 (0.821-4.660) | 0.130 |
| **HBcAb** |  |  |
| Negative | Reference | - |
| Positive | 0.528 (0.027-10.343) | 0.674 |

Data were analyzed in 134 patients in the training cohort with complete clinical information. All 10 prespecified clinical variables were entered simultaneously into the multivariable Cox model without prior univariable screening. P values are two-sided. Reference categories are indicated. AFP, alpha-fetoprotein; CI, confidence interval; HR, hazard ratio; MWA, microwave ablation; RFA, radiofrequency ablation.

**Supplementary Table S2.** Brier scores of the clinical, integrated radiomics, and clinical-radiomics combined models in the training and internal validation cohorts

| **Model** | **Dataset** | **n** | **Brier score** |
| --- | --- | --- | --- |
| CM | Training cohort | 134 | 0.1748 |
| CM | Internal validation cohort | 56 | 0.1885 |
| IRM | Training cohort | 142 | 0.1662 |
| IRM | Internal validation cohort (full) | 62 | 0.1916 |
| IRM | Internal validation subset | 56 | 0.1969 |
| CRM | Training cohort | 134 | 0.1565 |
| CRM | Internal validation cohort | 56 | 0.2007 |

The CM and CRM were evaluated in 134 training-cohort patients and 56 internal-validation patients with complete clinical data. The IRM was evaluated in the full training cohort (n = 142) and the full internal validation cohort (n = 62), and was additionally assessed in the 56-patient internal validation subset for direct comparison with the CM and CRM. Lower Brier scores indicate better overall probabilistic accuracy. CM, clinical model; CRM, clinical-radiomics combined model; IRM, integrated radiomics model.

**Supplementary Figures**

**Supplementary Figure S1**

**Supplementary Figure S1.** Calibration curves of the CM, IRM, and CRM in the training and internal validation cohorts. Panels A–C show the calibration curves of the CM, IRM, and CRM in the training cohort, respectively. Panels D–F show the corresponding calibration curves in the internal validation subset of 56 patients with complete clinical data. Panel G shows the calibration curve of the IRM in the full internal validation cohort (n = 62). The diagonal line indicates perfect agreement between the predicted and observed probabilities. CM, clinical model; IRM, integrated radiomics model; CRM, clinical-radiomics combined model.

**Supplementary Figure S2**

**Supplementary Figure S2.** Decision curve analyses of the clinical, integrated radiomics, and clinical-radiomics combined models. (A, B) Decision curve analyses comparing the CM, IRM, and CRM in the training cohort and the common 56-patient internal validation subset, respectively. (C–E) Decision curve analyses of the IRM in the training cohort, the 56-patient internal validation subset, and the full 62-patient internal validation cohort, respectively. In the plot legends, “clinical,” “image,” and “fused” correspond to the CM, IRM, and CRM, respectively.
